# Activation of Neutrophil Granulocytes by Platelet-Activating Factor Is Impaired During Experimental Sepsis

**DOI:** 10.3389/fimmu.2021.642867

**Published:** 2021-03-16

**Authors:** Stefan Hug, Stefan Bernhard, Alexander Elias Paul Stratmann, Maike Erber, Lisa Wohlgemuth, Christiane Leonie Knapp, Jonas Martin Bauer, Laura Vidoni, Michael Fauler, Karl Josef Föhr, Peter Radermacher, Andrea Hoffmann, Markus Huber-Lang, David Alexander Christian Messerer

**Affiliations:** ^1^Institute of Clinical and Experimental Trauma Immunology, University Hospital of Ulm, Ulm, Germany; ^2^Institute of General Physiology, University of Ulm, Ulm, Germany; ^3^Department of Anesthesiology and Intensive Care Medicine, University Hospital of Ulm, Ulm, Germany; ^4^Institute of Anesthesiological Pathophysiology and Process Development, University Hospital of Ulm, Ulm, Germany

**Keywords:** platelet-activating factor, neutrophil granulocytes, intracellular pH, sepsis, membrane potential, flow cytometry

## Abstract

Platelet-activating factor (PAF) is an important mediator of the systemic inflammatory response. In the case of sepsis, proper activation and function of neutrophils as the first line of cellular defense are based on a well-balanced physiological response. However, little is known about the role of PAF in cellular changes of neutrophils during sepsis. Therefore, this study investigates the reaction patterns of neutrophils induced by PAF with a focus on membrane potential (MP), intracellular pH, and cellular swelling under physiological and pathophysiological conditions and hypothesizes that the PAF-mediated response of granulocytes is altered during sepsis. The cellular response of granulocytes including MP, intracellular pH, cellular swelling, and other activation markers were analyzed by multiparametric flow cytometry. In addition, the chemotactic activity and the formation of platelet–neutrophil complexes after exposure to PAF were investigated. The changes of the (electro-)physiological response features were translationally verified in a human *ex vivo* whole blood model of endotoxemia as well as during polymicrobial porcine sepsis. In neutrophils from healthy human donors, PAF elicited a rapid depolarization, an intracellular alkalization, and an increase in cell size in a time- and dose-dependent manner. Mechanistically, the alkalization was dependent on sodium-proton exchanger 1 (NHE1) activity, while the change in cellular shape was sodium flux- but only partially NHE1-dependent. In a pathophysiological altered environment, the PAF-induced response of neutrophils was modulated. Acidifying the extracellular pH *in vitro* enhanced PAF-mediated depolarization, whereas the increases in cell size and intracellular pH were largely unaffected. *Ex vivo* exposure of human whole blood to lipopolysaccharide diminished the PAF-induced intracellular alkalization and the change in neutrophil size. During experimental porcine sepsis, depolarization of the MP was significantly impaired. Additionally, there was a trend for increased cellular swelling, whereas intracellular alkalization remained stable. Overall, an impaired (electro-)physiological response of neutrophils to PAF stimulation represents a cellular hallmark of those cells challenged during systemic inflammation. Furthermore, this altered response may be indicative of and causative for the development of neutrophil dysfunction during sepsis.

## Introduction

Platelet-activating factor (PAF) is a phospholipid mediator with well-described proinflammatory properties, among others, by activating thrombocytes and leukocytes (1, 2). The importance of PAF has been investigated in many other diseases, in particular in chronic inflammation including coronary artery disease, asthma, and rheumatoid arthritis, as well as in acute inflammation such as trauma and sepsis (1–4).

Sepsis is defined as life-threatening organ dysfunction caused by a dysregulated host response to infection (5). Upon overwhelming stimulation, the immune system becomes excessively activated, resulting in a dysfunctional immune response, which contributes to multi-organ dysfunction syndrome, and ultimately, lethality (5, 6). In this context, previous studies in cecal ligation and puncture-induced rodent sepsis reported some beneficial effects by inhibiting PAF activity (7). However, despite initial encouraging results in a phase II study (8, 9), PAF degradation (10) or administration of PAF receptor antagonists (11) failed to reduce lethality in patients with severe sepsis.

Neutrophils are the vanguard of innate cellular immunity being crucially involved in the clearance of pathogens. The activity of neutrophils is driven by many proinflammatory mediators including anaphylatoxins such as PAF and the complement cleavage product C5a, interleukins, microbe-associated molecular patterns (MAMPs, e.g., fMLF), and many other factors, activate neutrophils (6, 12–15). On a cellular level, neutrophils respond with membrane potential (MP) depolarization likely generated by NADPH oxidase (NOX) activity (16, 17). Additionally, the intracellular pH (pH_i_) of neutrophils increases transiently (15, 18), regulating important cellular functions, for example, interference of apoptosis (19, 20), and modulation of chemotactic activity (21–23). Moreover, neutrophil activation by chemoattractants results in changes in the cellular shape and chemotaxis (14, 24), the generation of reactive oxygen species (ROS) (25, 26), and the expression of surface activation markers. For the last, an upregulation of integrin alpha M (CD11b) was found to facilitate leukocyte adhesion and L-selectin (CD62L) shedding, which both are considered to be hallmarks of neutrophil diapedesis into peripheral tissues (27–29).

The PAF-induced response in various cell types, including neutrophils, was investigated previously. For example, PAF induced a depolarization in rat endothelial cells (30), guinea pig neurons (31), and human neutrophils (32). Moreover, PAF mediated a small increase in pH_i_ in bovine neutrophils (33, 34), while in human neutrophils an initial acidification and consecutive small rebound alkalization was described (35). During systemic inflammation, cellular parameters of neutrophils have been reported to be altered. For example, in murine sepsis, an increase in neutrophil cell size (14) and pH_i_ were reported, with the latter verified in patients with sepsis (15). Both alterations have been confirmed in an *ex vivo* model of lipopolysaccharide (LPS)-induced inflammation (36). In this context, it is tempting to speculate that a shift in baseline levels of neutrophil parameters during systemic inflammation affects the response under additional stimulation by inflammatory mediators such as PAF, however, this remains to be further elucidated.

Therefore, in this study, we investigated the cellular response induced by PAF in neutrophils in a multi-step approach: Firstly, under physiological conditions and, secondly mechanistically by identifying the sodium-proton antiport as an important ion transport protein. Finally, we translationally analyzed the response elicited by PAF during inflammation in the setting of *in vitro* acidosis, in an *ex vivo* whole blood model of LPS-driven inflammation, and in a porcine model of polymicrobial sepsis.

## Materials and Methods

All chemicals were purchased from Merck (Darmstadt, Germany), when not indicated otherwise.

### Isolation of Neutrophils

The investigations were approved by the Local Independent Ethics Committee of the University of Ulm (number 459/18; 94/14). After obtaining informed written consent from healthy human volunteers, blood was drawn into syringes containing 3.2% trisodium citrate (Sarstedt, Nürnbrecht, Germany). Neutrophils were isolated by Ficoll-Paque (GE Healthcare, Uppsala, Sweden) density gradient centrifugation and subsequent dextran sedimentation followed by hypotonic lysis of remaining erythrocytes, as described previously (14–16). Polymorphonuclear granulocytes (mainly consisting of neutrophils) were adjusted to a concentration of 2 × 10^6^ cells/ml using Hank's balanced salt solution with calcium and magnesium (HBSS, Thermo Fisher, Darmstadt, Germany), the pH of which was adjusted to 7.3, when not indicated otherwise.

### Measurement of Neutrophil Membrane Potential, Intracellular pH, and Cell Size

Isolated neutrophils were incubated in a light-protected water bath at 37°C with the fluorescent dyes bis(1,3-dibutylbarbituric acid) trimethine oxonol (DiBAC_4_(3)), 50 nM, or 5-(and-6)-carboxy-SNARF-1 (SNARF), 1 μM (Thermo Fisher) for 20 min or with dihydrorhodamine 123 (D123) (Santa Cruz Biotechnology, Heidelberg, Germany), 1.8 μM, for 30 min to determine MP (16), pH_i_, and ROS generation [what is measured by D123 is the byproduct H_2_O_2_ which is itself derived from ${\text{O}}_{2}^{•-}$, a product generated by NOX activity (37)], respectively. To determine porcine neutrophil pH_i_, the cells were instead incubated with 2′,7′-bis-(2-carboxyethyl)-5-(and-6)-carboxyfluorescein, acetoxymethyl ester (BCECF, Abcam, Cambridge, United Kingdom), 25 nM, for 30 min, because exposure of porcine neutrophils to SNARF resulted in changes in forward scatter area (FSC-A), which was interpreted as possible cellular activation (data not shown). Following incubation, neutrophils were centrifuged for 5 min (340 g) and resuspended in Roswell Park Memorial Institute medium (RPMI) with magnesium and calcium with a pH adjusted to 7.3. DiBAC_4_(3) was again added. After a resting period of 10 min in a light-protected water bath at 37°C, neutrophils were stimulated with 1 μM PAF or 100 ng/ml complement factor 5a (C5a, Complement Technology, Tyler, Texas, USA), when not indicated otherwise. Cells were analyzed using a Canto II flow cytometer (BD Biosciences, Heidelberg, Germany). Neutrophils were identified by FSC-A and sideward scatter area. Non-fluorescent reference microspheres (10, 15, and 20 μm diameter; Polybead® Polystyrene Microsphere, Polysciences, Hirschberg, Germany) were used to quantify the shape changes assessed by the FSC-A (14). Differences in absolute FSC-A values between experiments are explained by recalibration of the flow cytometer between different experimental series. The fluorescence of DiBAC_4_(3) were converted in changes of the MP as described previously (16) with a small modification: RPMI with varying extracellular concentrations of sodium and potassium were used to calculate the amount of change in DiBAC_4_(3) fluorescence per mV.

Near-real time kinetics were measured by acquiring neutrophils for 10 min continuously embedded in a heating unit (TC-1234A Temperature Controller, Warner Instruments LLC, Holliston, Massachusetts, USA), ensuring a steady temperature level of 37°C. Analysis was performed by a specifically designed algorithm using the statistic language R (R Core Team, Vienna, Austria).

### Cell Surface Markers

Isolated neutrophils were incubated in a light-protected water bath at 37°C. Following PAF stimulation as indicated above (normally 1 μM) for 10 min, neutrophils were stained with 0.1 μg/ml APC anti-mouse/human CD11b antibody (#101212, Biolegend, San Diego, USA) and 0.5 μg/ml PE anti-human CD62L antibody (#304806, Biolegend) for 5 min at room temperature. Cells were fixed using FACS Lysing Solution (BD, Heidelberg, Germany). Proper isotype controls were used (CD11b: APC Rat IgG2b, κ Isotype Ctrl; CD62L: PE Mouse IgG1, κ Isotype Ctrl; Biolegend).

### *In vitro* Pharmacological Modification

PAF-induced effects on neutrophils were analyzed in the presence of the subsequently listed pharmacological modulators (their proposed targets are given in brackets): Amiloride [200 μM, 10 min, Na^+^-channels (15)], BIX [5 μM, 30 min, sodium-proton exchanger 1 (NHE1) (38); Tocris, Wiesbaden, Germany], 5-Nitro-2-(3-phenylpropylamino)benzoic acid [NPPB, 100 μM, 10 min, Cl^−^ channels (39)], and ebselen [10 nM, 30 min, glutathione peroxidase and peroxiredoxin enzyme mimetic and NOX2 inhibitor (40, 41); Tocris]. Following pre-incubation, samples were divided to obtain paired results and treated as control or stimulated with PAF.

### Extracellular Alkalosis and Acidification *in vitro*

Following the described staining and centrifugation, neutrophils were resuspended in RPMI adjusted to a pH of 6.6, 7.0, 7.4, or 7.8 to simulate extracellular alkalosis or acidification. Neutrophils were incubated for 10 min at 37°C and subsequently stimulated with PAF.

### Coulter Counter Measurements

Isolated neutrophils were stimulated for 10 min, diluted 1:500 with RPMI adjusted to a pH 7.3 and measured by a cell counter working through electronic current exclusion (Cell Counter CASY, OLS OMNI Life Science, Bremen, Germany).

### Chemotaxis

Neutrophil chemotactic activity was assessed using a Neuro Probe A96 chemotaxis chamber (Neuro Probe, Gaithersburg, Maryland, USA). Isolated neutrophils at 5 × 10^6^ cells/ml in HBSS + 0.1% bovine serum albumin (BSA) were stained with the fluorescent dye BCECF (1.6 μg/ml) for 30 min at 37°C, subsequently centrifuged for 5 min (340 g), and resuspended in HBSS + 0.1% BSA. A total of 33 μl chemoattractant PAF (final concentration 1 μM) was added into the wells of the lower plate. Thereafter, a silicone gasket and a framed filter with 3 μm pores were placed upon the lower wells. On top of it, the upper plate was attached, and the dyed neutrophils were pipetted into the corresponding wells. During incubation for 30 min at 37°C, neutrophils migrated from the upper wells toward the lower wells containing PAF, but became adherent to the filter, resulting in increased fluorescence. The fluorescence of the cells in the filter was measured at a wavelength of 485/538 nm using a Fluoroskan Ascent (Thermo Scientific, Rockford, Illinois, USA) with the Ascent Software Version 2.6.

### Quantification of Platelet-Neutrophil Complex Formation

Platelet–neutrophil complex (PNC) formation, defined as a neutrophil with at least one platelet in direct proximity, was assessed as described previously by light microscopy and flow cytometry (36, 42). For blood smears, whole blood was diluted 1:1 with phosphate-buffered saline containing calcium and magnesium (PBS) and incubated for 15 min at 37°C with or without 1 μM PAF on a spinning wheel (Snijders Labs, Tilburg, Netherlands) at 3 rpm. Blood smears were created and stained with Hemacolor® Rapid staining (Merck). In each sample, 50 neutrophils were counted by two independent and blinded individuals. For flow cytometry analysis, whole blood was diluted 1:5 with PBS. Following 15 min of incubation with or without 1 μM PAF, the sample was stained with anti-CD41 APC (125 ng/ml, #303710, Biolegend) and anti-CD61 PerCP (250 ng/ml, # 336412, Biolegend; both monoclonal mouse anti-human antibodies) for 15 min at room temperature followed by 30 min incubation with 1 ml of 1X BD FACS Lysing solution (BD Biosciences, San Jose, California, USA). Samples were centrifuged for 5 min at 340 g and resuspended in 100 μl PBS + 0.1% BSA and stored at 4°C in the dark until further analysis (normally within 1 h).

### *Ex vivo* Human Whole Blood Model of Endotoxemia

An animal-free human whole blood model of endotoxemia was used to investigate the effect of LPS exposure on the PAF-induced response of neutrophils, which was described previously in detail (36). In brief, 0.5 IU/ml heparin (B. Braun Melsungen AG, Melsungen, Germany) and 100 ng/ml LPS (#L2630, Merck) or PBS (control) were added to 9 ml blood, which was transferred into a Cortiva BioActive Surface (Medtronic, Meerbusch, Germany) coated tubing system using a coated connector (Medtronic) to interlink both ends. Following 1 h of rotation (3 rpm) on a spinning wheel (Snijders Labs) at 37°C, the blood was transferred to citrate anti-coagulated monovettes (Sarstedt). Neutrophil activation markers were analyzed directly in whole blood as described previously (36) with or without exposure to PAF (1 μM, 15 min, 37°C). The remaining whole blood was processed to isolate and to analyze neutrophils as described above.

### Porcine Polymicrobial Sepsis

The Federal authorities for animal research (#1362, Tuebingen, Germany) as well as the Animal Care Committee of the University of Ulm approved the experiments. The well-described porcine sepsis model was performed in adherence with guidelines on the Use of Laboratory Animals of the National Institutes of Health with anesthesia and surgical instrumentation as described previously (43–45). In brief, nine Bretoncelles-Meishan-Willebrand pigs (5 male-castrated, 4 female, mean weight 65.6 kg ± 8.6) were subjected to polymicrobial sepsis induced by inoculation of autologous feces into the abdominal cavity, followed by intensive care therapy for 60 h after the sepsis initiation. Blood was drawn before inoculation and when the mean arterial pressure was reduced by >10%, indicating cardiocirculatory shock and triggering the beginning of resuscitation. To reduce animal numbers, the analyzed pigs are a subgroup of another currently unpublished trial comparing standard intensive care therapy with or without a pharmacological intervention targeting the calcitonin gene-related peptide receptor. Because intervention started after the fulfillment of the sepsis criteria listed above, animals were included irrespective of their group allocation.

### Data Presentation and Statistical Analysis

Results are presented as mean ± standard deviation (SD), when not indicated otherwise. In all experiments, a minimum of 3,000 neutrophils were measured. All data were considered to be paired and non-parametric. Statistical significance is indicated by ^*^, ^**^, and ^***^, with significance levels of *p* < 0.05, < 0.01, and < 0.001, respectively. Statistical analysis was performed using GraphPad Prism 9 (GraphPad Software Inc., San Diego, California, USA) and Microsoft Excel (Version 16.42, Microsoft Corporation, Redmond, Washington, USA).

## Results

### PAF Induced Changes in Neutrophil Physiology in a Dose- and Time-Dependent Manner

**Figure 1:**
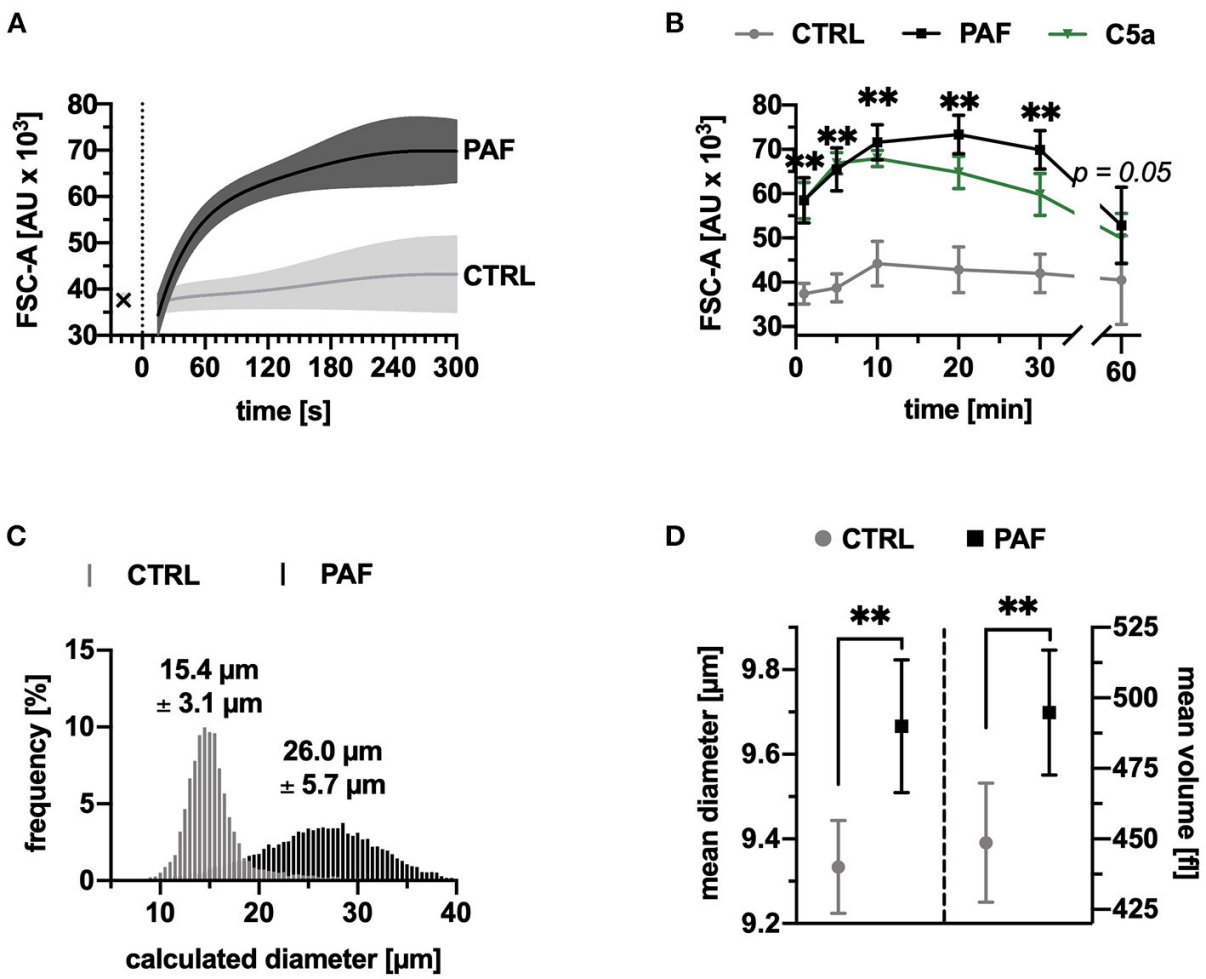
PAF elicited a rapid increase in size of human neutrophils. **(A)** FSC-A (forward scatter area) of neutrophils stimulated by PAF (1 μM, black) or PBS (CTRL, gray), measured by continuous acquisition by flow cytometry for 5 min, x indicating baseline measurement, *n* = 5. **(B)** FSC-A of neutrophils stimulated with PAF or PBS for 60 min, *n* = 15. For comparison, neutrophils exposed to C5a (green) are shown. **(C)** Calculated diameter of neutrophils using counting beads (10, 15, and 20 μm) from one representative donor with or without PAF exposure. **(D)** Mean diameter and volume of neutrophils stimulated by PAF or PBS measured by Coulter counter, *n* = 8. Data are mean ± SD. **p < 0.01, Wilcoxon matched-pairs signed rank test comparing PAF-stimulated neutrophils with unstimulated control cells.

Neutrophils responded to PAF stimulation with a rapid increase in cell size, peaking after 20 min with a relative increase of 73 ± 19% (Figures 1A,B). A near-real time measurement demonstrated that most of the size change occurred within in the first minutes (Figure 1A). The increase in the FSC-A induced by PAF was similar to the stimulation with C5a (Figure 1B). Using reference microspheres, the calculated diameters of unstimulated cells were 16.9 ± 1.1 μm, and 26.1 ± 0.6 μm for PAF-stimulated cells (after 10 min, *p* = 0.06, *n* = 5, representative measurement in Figure 1C). The change in neutrophil size was verified by Coulter counter measurement. Following 10 min of incubation with PAF, neutrophils exhibited an increase of 3.6 ± 1.7% in diameter and 10.5 ± 6.4% in volume in comparison with unstimulated neutrophils (Figure 1D).

**Figure 2:**
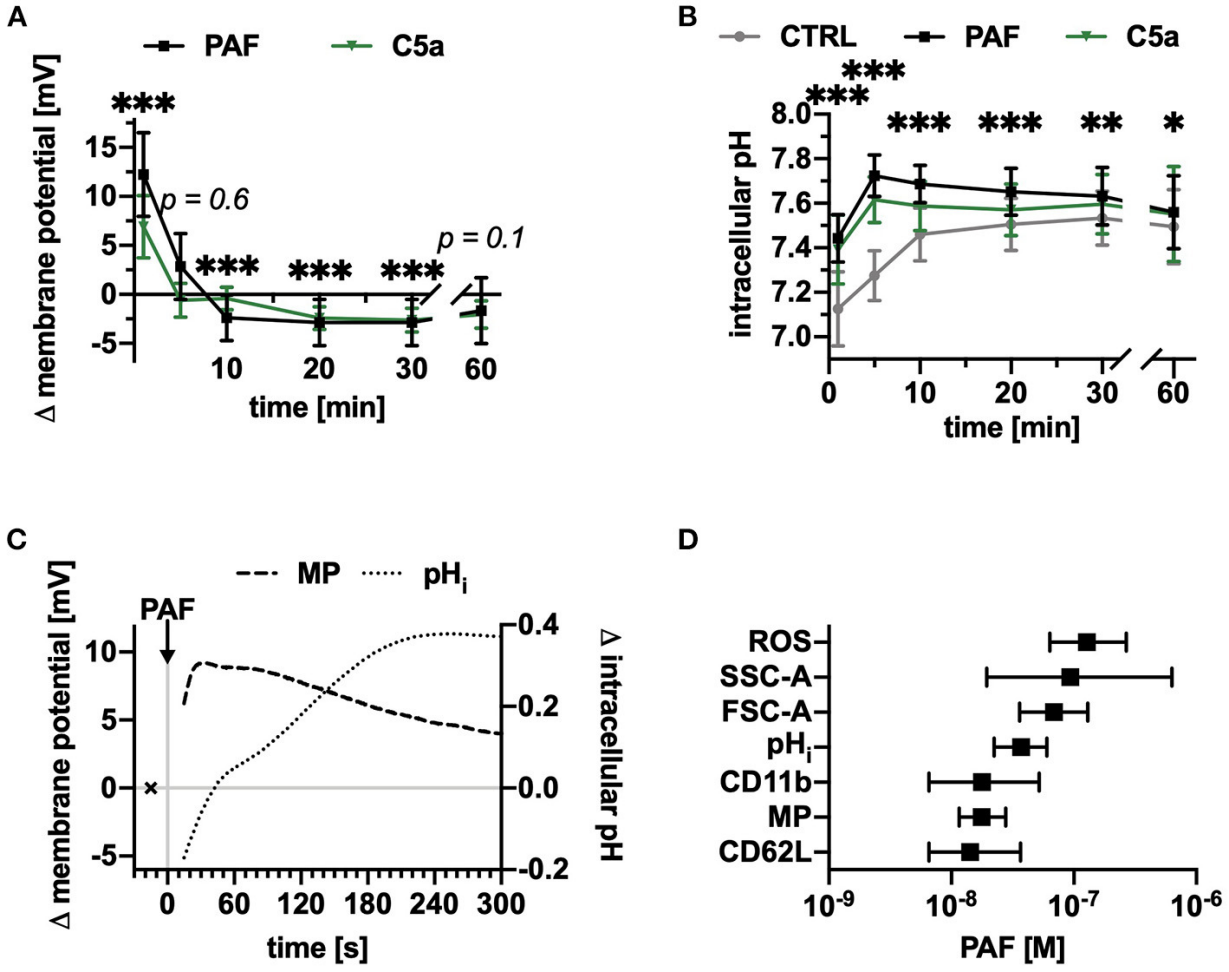
PAF depolarized the membrane potential and increased the intracellular pH of human neutrophils within seconds. **(A)** Changes in membrane potential of neutrophils stimulated with PAF (1 μM) or C5a (green, 10 nM), *n* = 15, unstimulated cells = 0 mV. **(B)** Intracellular pH of neutrophils upon stimulation with PAF, C5a, or PBS (CTRL), *n* = 15. **(C)** Changes in membrane potential and intracellular pH of neutrophils exposed to PAF measured by continuous acquisition on flow cytometry. x represents the baseline measurement prior to stimulation. Depicted is one representative donor out of five independent experiments. **(D)** PAF-induced effects on neutrophils were concentration-dependent (ROS, reactive oxygen species; SSC-A, side scatter area; FSC-A, forward scatter area; pH_i_, intracellular pH; CD11b, integrin alpha M; MP, membrane potential; CD62L, L-selectin) and respective EC_50_ ± interquartile range were calculated (*n* = 5–10). **(A,B)** data are mean ± SD. *p < 0.05, **p < 0.01, ***p < 0.001, Wilcoxon signed-rank test **(A)** and Wilcoxon matched-pairs signed rank test **(B)** comparing PAF-stimulated neutrophils with unstimulated control cells.

Next, the response elicited by PAF on the neutrophil MP and pH_i_ was investigated. PAF rapidly induced a depolarization peaking after 1 min (Figure 2A) and an intracellular alkalization attaining a maximum of +0.45 ± 0.07 after 5 min (Figure 2B). In addition, PAF induced an increase in ROS production (+26 ± 11% after 10 min, *p* < 0.001 Wilcoxon signed-rank test, *n* = 17, data not shown). Depolarization and intracellular alkalization revealed different kinetics (Figures 2A–C). The PAF-induced response was comparable to C5a-stimulation for cell size and pH_i_ but was more intensive for the MP (1 min: PAF +12 ± 4 mV, C5a: 7 ± 3 mV, Figures 2A,B). All PAF-induced effects showed a clear concentration-response relationship (Supplement 1). The EC_50_-values are presented in Figure 2D and ranged between 14 and 128 nM.

**Figure 3:**
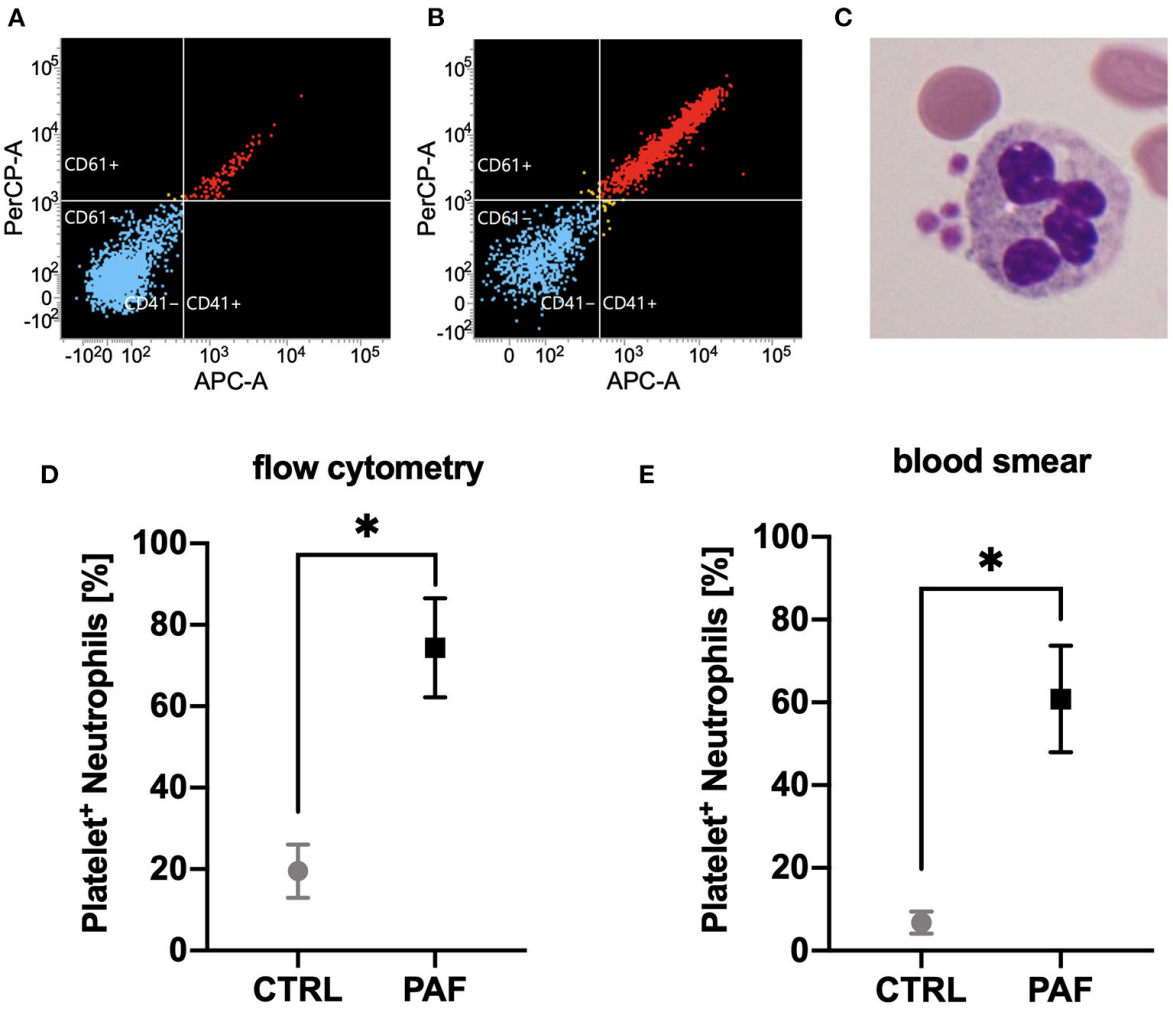
PAF mediated the formation of platelet–neutrophil complexes (PNCs). **(A,B)** Flow cytometric measurements of PNCs using APC-labeled anti-CD41 and PerCP-labeled anti-CD61 antibodies. Shown is one representative donor for blood stimulated with PBS (CTRL, **A**) or PAF 1 μM **(B)**. Blue indicates CD41- and CD61-negative cells, yellow indicates either CD41- or CD61-positive neutrophils, red refers to neutrophils positive for CD41 and CD61. **(C)** Light microscopy of an exemplary PNC. **(D)** Quantification of PAF-induced (1 μM) PNC formation by flow cytometry (*n* = 7). **(E)** PAF mediated PNC formation as detected by blood smear and manual counting (*n* = 6). Data are mean ± SD. *p < 0.05, Wilcoxon matched-pairs signed rank test.

In accordance with previous findings, neutrophils responded to PAF stimulation with an increased chemotactic activity (15.8 ± 9.7 fold increase after 30 min, *p* < 0.05 Wilcoxon signed-rank test, *n* = 6), CD11b upregulation (+63.3 ± 41.9% after 10 min, *p* < 0.01 Wilcoxon signed-rank test, *n* = 8), and CD62L shedding (−87 ± 10% surface expression after 10 min, *p* < 0.01 Wilcoxon signed-rank test, *n* = 8) (Supplements 1, 2). In addition, PAF induced formation of PNCs (Figure 3). For all these effects, there was no significant difference when comparing neutrophils from male and female donors (Supplement 2).

### Ion Transport Proteins and NOX2 Modulated the PAF-mediated Neutrophil Response

**Figure 4:**
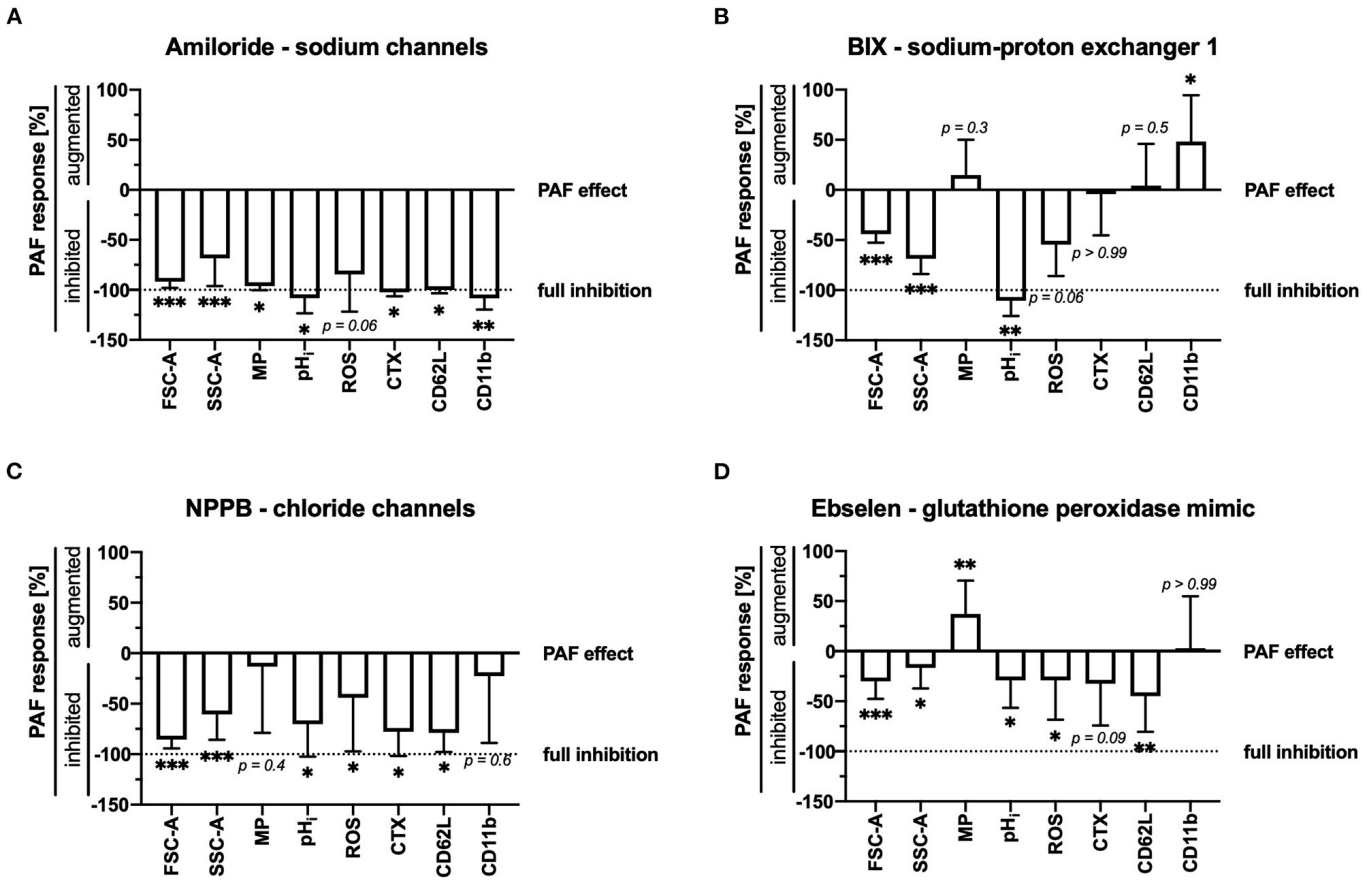
**(A)** Amiloride (200 μM), **(B)** BIX (5 μM), **(C)** NPPB (100 μM), and **(D)** ebselen (10 nM) modulated the PAF-mediated response of neutrophils. While 0% indicates the regular response induced by 1 μM PAF, −100% indicates full inhibition of the PAF-mediated response = control level. For the calculation, see Supplement 3. Data are mean ± SD (*n* = 5–12). *p < 0.05, **p < 0.01, ***p < 0.001, Wilcoxon signed-rank test.

Ion fluxes in stimulated neutrophils are crucially involved in cellular activity. Unspecific inhibition of sodium or chloride flux inhibited most cellular changes induced by PAF (Figures 4A,C), however, in parallel also partially altering unstimulated neutrophils (Supplement 3).

A more detailed analysis revealed that PAF-induced intracellular alkalization was completely inhibited by targeting NHE1 activity with BIX (+0.30 ± 0.07 vs. −0.02 ± 0.04, *p* < 0.01). Cellular swelling and the ROS generation were also reduced, but to a lesser extent, by targeting NHE1 (Figure 4B). PAF-induced depolarization and CD62L shedding displayed no difference during selective NHE1 inhibition. PAF-induced CD11b expression increased during selective NHE1 inhibition, whereas it was fully inhibited by amiloride. However, during incubation with the NHE1 inhibitor BIX, neutrophils expressed less CD11b than cells without an inhibitor (Supplement 3).

Mimicking glutathione peroxidase activity and inhibiting NOX2 with ebselen significantly lowered PAF-induced generation of intracellular alkalization, ROS generation and CD62L shedding (Figure 4D).

### Acidosis Significantly Enhanced PAF-mediated Depolarization

**Figure 5:**
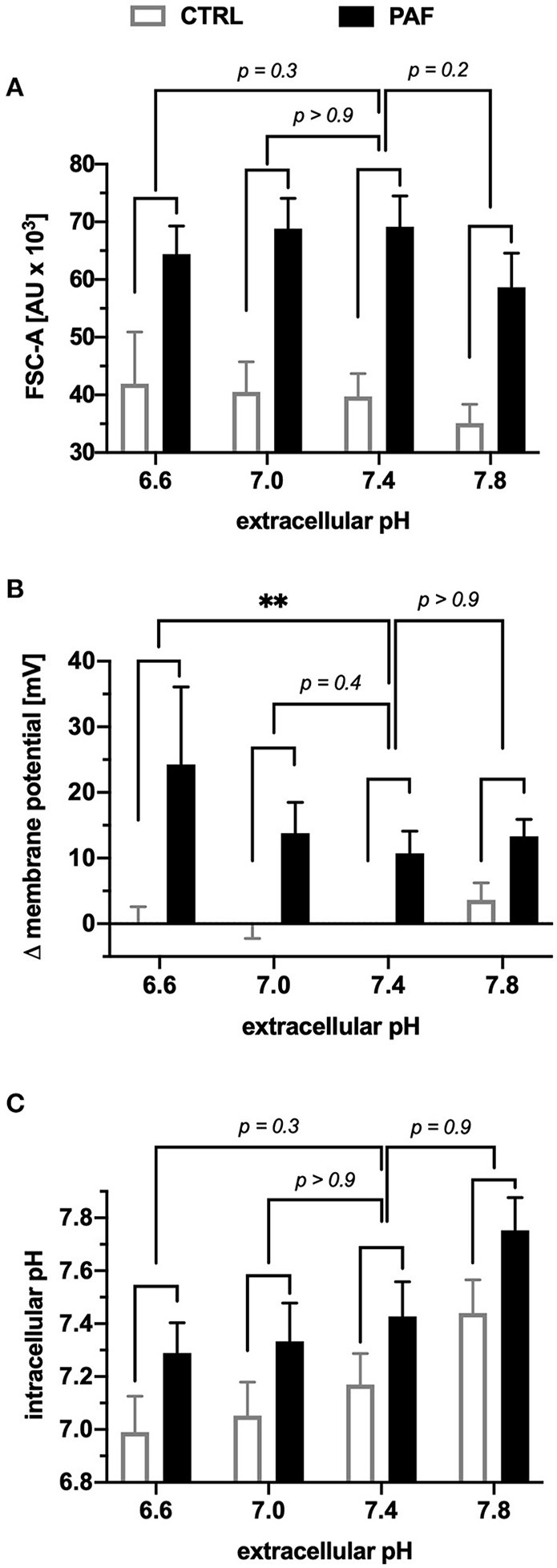
Extracellular acidosis increased the PAF-mediated depolarization in human neutrophils *in vitro*. Neutrophils were incubated in buffers with different extracellular pH and subsequently stimulated by 1 μM PAF. **(A)** The increase in the FSC-A induced by PAF in comparison with unstimulated neutrophils in an acidotic or alkaline environment did not significantly alter the cellular response in comparison to an extracellular pH of 7.4. **(B)** Depolarization of the membrane potential upon PAF stimulation was enhanced in an acidic environment (0 mV = CTRL extracellular pH 7.4). **(C)** Intracellular pH of neutrophils stimulated with PAF (black) or PBS (CTRL, white). Data are mean ± SD (*n* = 6). **p < 0.01, Kruskal Wallis and Dunn's *post-hoc* test comparing the PAF-induced response for each extracellular pH.

The first step to investigate the above-mentioned PAF-elicited effects under pathologic conditions was to expose neutrophils to buffers of various extracellular pH (pH_e_). The pH_e_ had no influence on the PAF-induced increase in the FSC-A (Figure 5A). Depolarization after PAF stimulation was significantly enhanced under acid extracellular conditions (pH_e_ 6.6 = +23.6 ± 7.5 mV vs. pH_e_ 7.4 = +10.7 ± 3.1 mV, *p* < 0.01 Kruskal-Wallis test and Dunn's multiple comparison test, *n* = 6, Figure 5B). Regarding the pH_i_, neutrophils responded with an equal intracellular alkalization when stimulated with PAF under different extracellular conditions. In addition, the pH_i_ of neutrophils shifted with the pH_e_, therefore with rising pH_e_, the pH_i_ increased in parallel (Figure 5C).

### Endotoxemia Modulated the PAF-induced Response of Neutrophils

**Figure 6:**
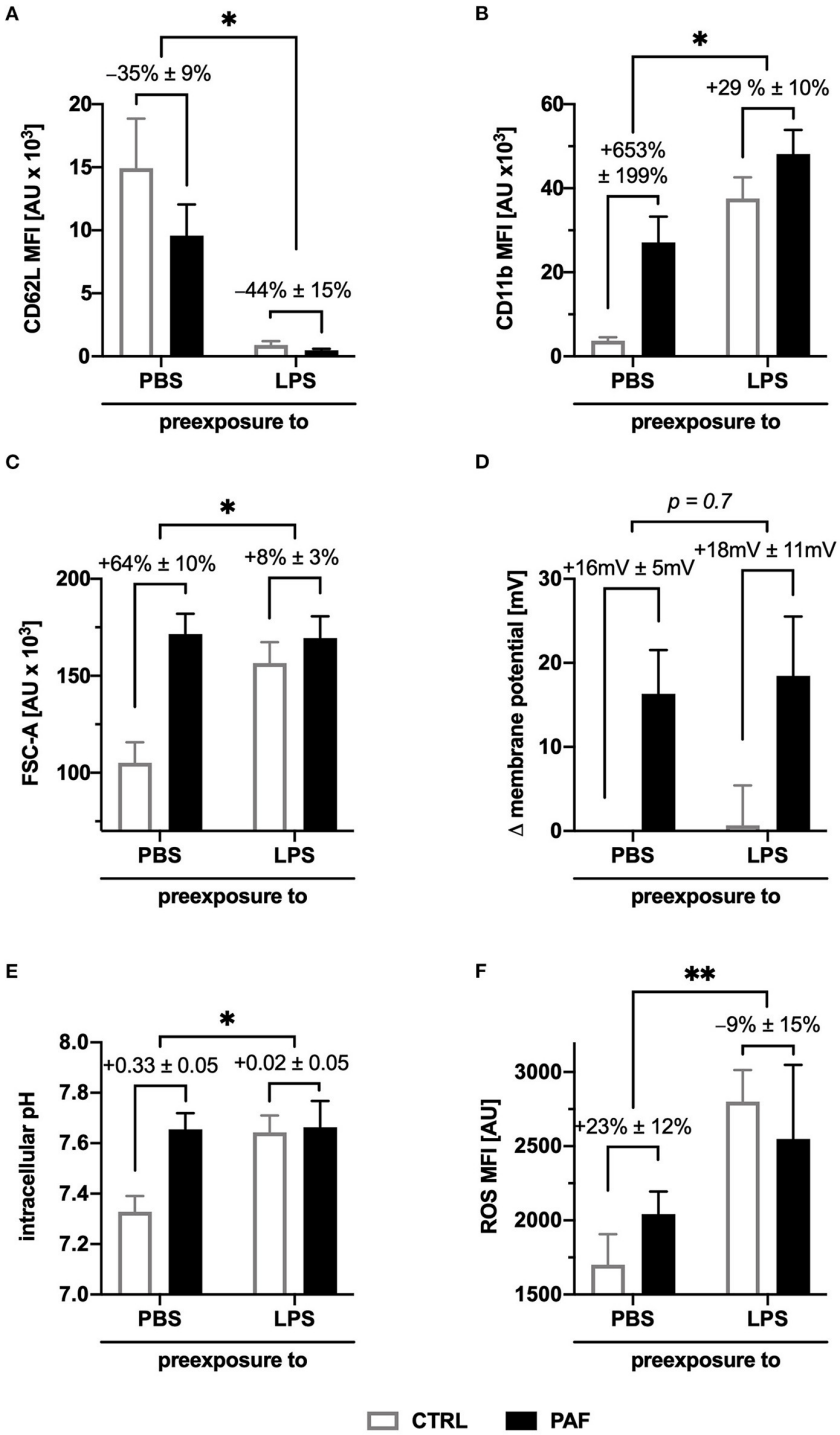
LPS-driven inflammation altered the PAF-induced response pattern of human neutrophils in an *ex vivo* whole blood model. Blood was incubated for 1 h with or without 100 ng/ml LPS. Subsequently, blood **(A,B,F)** or isolated neutrophils **(C–E)** were stimulated with 1 μM PAF *in vitro*. **(A,B)** Surface expression of L-selectin (CD62L, **(A)**) and integrin alpha M (CD11b, **(B)**) on neutrophils. **(C)** Cellular size of neutrophils as indicated by the FSC-A. **(D)** PAF-induced depolarization after exposure to LPS. **(E)** Intracellular pH of neutrophils stimulated with or without PAF. **(F)** PAF-mediated ROS generation. Data are mean ± SD of the PAF-induced response (*n* = 6). *p < 0.05, **p < 0.01, Wilcoxon matched-pairs signed rank test comparing the response induced by PAF of neutrophils with (LPS) or without (PBS as CTRL) stimulation in the whole blood model. MFI, mean fluorescence intensity.

To translate the findings to a more clinically relevant setting, an *ex vivo* model of human endotoxemia was used analyzing the modulation of the PAF-induced response in systemic inflammation. As previously reported (36), sole contact with the tubing system of the model did not promote neutrophil activation (data not shown). However, the exposure to LPS (100 ng/ml) resulted in an activated neutrophil phenotype as reflected by of the upregulation of the surface expression of CD11b and downregulation of CD62L. These alterations in baseline expression of these activation markers also modulated the response generated by additional *in vitro* stimulation with PAF (increase in CD62L downregulation, reduction in CD11b upregulation, Figures 6A,B).

LPS exposure resulted in increased cellular size, pH_i_, and generation of ROS, but not depolarization in neutrophils without further PAF stimulation. The PAF-induced increase in cellular size was significantly reduced after exposure to LPS (CTRL-PAF +64 ± 10% vs. LPS-PAF +8 ± 3%, *p* < 0.05, Figure 6C). There was no significant difference in PAF-mediated depolarization after exposure to LPS (Figure 6D). Transient intracellular alkalization elicited by PAF was significantly reduced in neutrophils after endotoxemia (CTRL-PAF +0.33 ± 0.05 vs. LPS-PAF +0.02 ± 0.05, *p* < 0.05, Figure 6E). The PAF-induced elevation in ROS generation was reduced after LPS exposure (CTRL-PAF +23 ± 12% vs. LPS-PAF −9 ± 15%, Figure 6F).

### Sepsis Partially Impaired PAF-induced Neutrophil Activation

**Figure 7:**
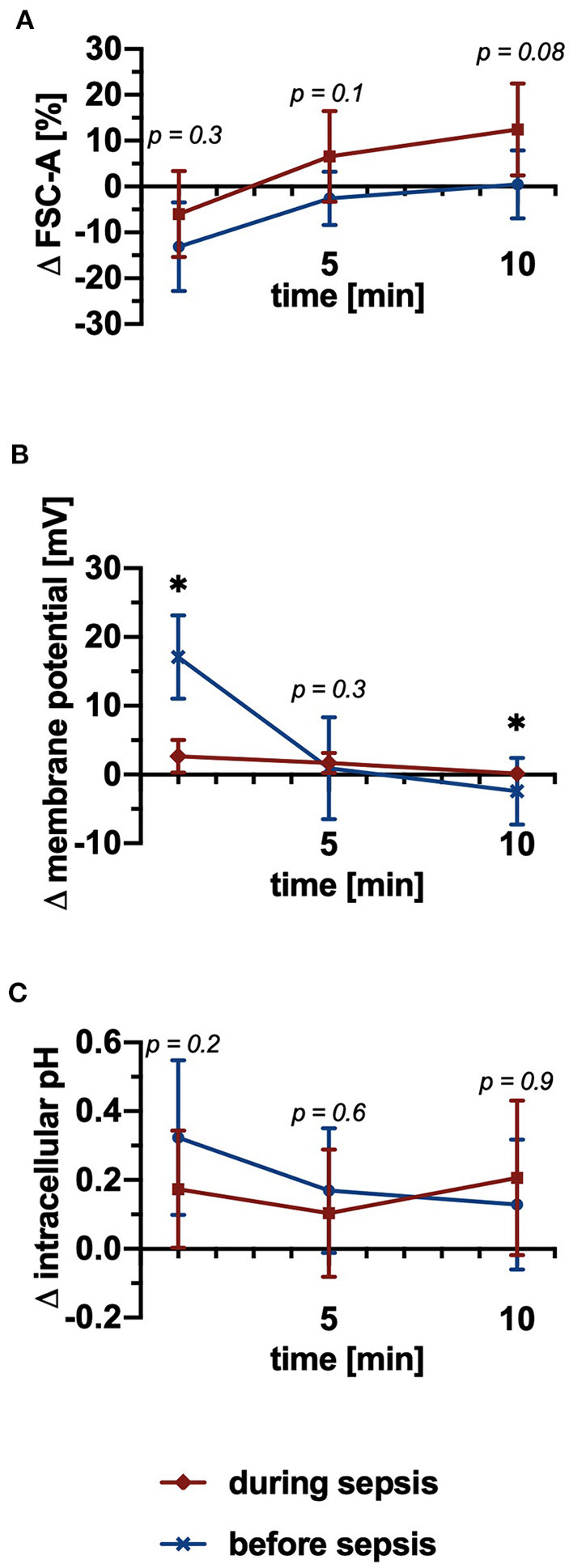
PAF-mediated depolarization was significantly impaired and there was a trend for increased cellular swelling during experimental polymicrobial sepsis, whereas alkalization remained stable. **(A)** Changes of the FSC-A upon stimulation with 1 μM PAF vs. unstimulated cells before (blue) and during (red) sepsis (*n* = 9). **(B)** PAF-induced alteration in membrane potential (*n* = 7). **(C)** Alterations in intracellular pH of PAF-exposed neutrophils (*n* = 9). Data are mean ± SD. *p < 0.05, Wilcoxon matched-pairs signed rank test.

To validate the *in vitro* and *ex vivo* results, neutrophils isolated from a porcine polymicrobial sepsis model were investigated before and during sepsis. Clinical parameters and electrolytes are summarized in Table 1. The baseline FSC-A and pH_i_ of neutrophils without PAF stimulation remained stable before and during sepsis (data not shown). The PAF-induced change in cellular shape displayed a trend for an increase during sepsis (+0 ± 7% before vs. +12 ± 10% during sepsis, *p* = 0.08, Figure 7A). PAF-mediated depolarization was significantly diminished during sepsis (+17.1 ± 5.6 mV before vs. +2.6 ± 2.2 mV during sepsis, *p* < 0.05, Figure 7B). There was no difference between neutrophils before and during sepsis regarding the PAF-induced intracellular alkalization (Figure 7C).

**Table 1:** Clinical parameters of the animals before and during experimental polymicrobial sepsis.

**Parameter**	**Unit**	**Before sepsis**	**During sepsis**	***p*-value**
Heart rate	per min	103 ± 19	170 ± 32	^*^
MAP	mmHg	104 ± 9	82 ± 9	^*^
Hb	g/dl	8.6 ± 0.6	14.5 ± 3.7	^**^
pH	–log(H^+^)	7.51 ± 0.04	7.48 ± 0.04	0.08
Lactate	mM	1.4 ± 0.5	1.8 ± 1.1	0.30
K^+^	mM	3.0 ± 0.2	3.3 ± 0.2	^*^
Na^+^	mM	143.7 ± 2.7	143.8 ± 2.6	>0.99
Ca^2+^	mM	0.73 ± 0.06	0.69 ± 0.09	0.64
Time	h	–	5.8 ± 3.5	–

*MAP, mean arterial pressure; Hb, hemoglobin concentration; K^+^, blood potassium concentration; Na^+^, blood sodium concentration; Ca^2+^, blood calcium concentration; Time, time from initiation to decrease in MAP indicating sepsis. Data are mean ± SD (n = 9)*.

* *p < 0.05*,

** *p < 0.01 Wilcoxon matched-pairs signed rank test*.

## Discussion

### PAF-induced Depolarization, Alkalization, and Cellular Swelling Under Physiological Conditions

**Figure 8:**
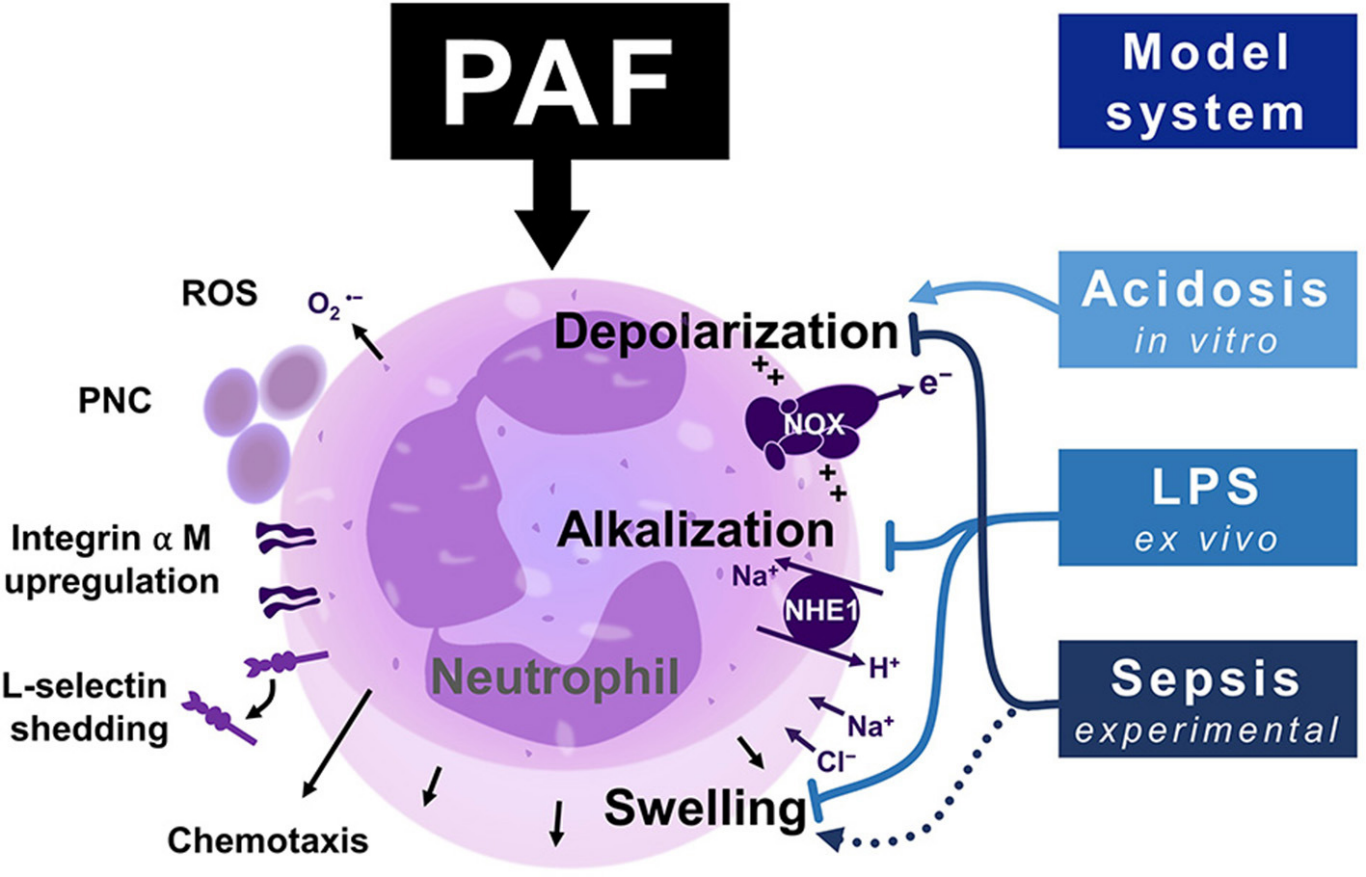
Graphical summary of the neutrophil response upon exposure to platelet-activating factor (PAF), including depolarization, NHE1-dependent alkalization, and cellular swelling and functional thromboinflammatory activity. The (electro-)physiological response mediated by PAF is modulated in inflammatory environments. Endotoxemia increases intracellular pH and cell size of neutrophils while in parallel diminishing an additional change inducible by PAF. Depolarization is enhanced by extracellular acidosis, while being significantly disrupted during experimental sepsis. ROS, reactive oxygen species; PNC, platelet–neutrophil complex; NOX, NADPH oxidase; NHE1, sodium-proton exchanger 1.

The inflammatory mediator PAF induced a rapid sequence of cellular reactions in neutrophils in a concentration- and time-dependent manner as summarized in Figure 8. The initial depolarization within seconds can likely be explained by the activation of the NOX activity resulting in excessive electron extrusion (17). This is corroborated by increased ROS generation and the lack of cellular depolarization in patients with a NOX defect (46). Depolarization was followed by intracellular alkalization within minutes. The time course of the PAF-induced alkalization was in accordance with reports from bovine neutrophils (33). The change in pH_i_ elicited by PAF was less in the study by Hidalgo et al., however, this might result from the use of different dyes and/or species differences [(33) and the results presented in this work]. In this context, it is noteworthy that shifts in neutrophil pH_i_ are associated with other cellular functions, for example increasing chemotactic motility (21, 23, 47). Therefore, the impact of PAF-mediated transient alkalization requires further elucidation.

In parallel, neutrophils responded to PAF stimulation with an increase in cellular size as indicated by FSC-A. The rise in cell size was confirmed by analyzing the cell volume by applying the coulter counter principle, although with less pronounced changes. In this context, previous studies with C5a indicated that an increased FSC-A reflects a cellular elongation (14, 48) likely caused by actin polymerization (48). This possibly explains the differences between the PAF-induced changes in cell size reported by different methods. Overall, the PAF-mediated response was similar to that elicited by other chemoattractants, including the complement-derived anaphylatoxin C5a (14–16) and fMLF (18, 35, 46).

The cellular response of neutrophils induced by PAF was also investigated in the absence or presence of pharmacological inhibitors with a special interest in depolarization, intracellular pH, and swelling. In accordance with the results obtained on neutrophil stimulation with other chemokines (14, 15, 33), non-specific inhibition of sodium flux with amiloride and/or chloride flux with NPPB significantly reduced the intracellular alkalization and cellular swelling. Albeit amiloride also reduced depolarization, it should be noted that it also pre-depolarized unstimulated cells. Next, we investigated the role of NHE1 as an important representative ion transporter protein in stimulated granulocytes (15, 38, 49). The pharmacological NHE1 inhibitor BIX (38, 50) also inhibited intracellular alkalization and to a lesser extent, cellular swelling, but not early depolarization. This finding of the PAF-neutrophil interaction is in accordance with previous reports for fMLF and C5a (15, 23), as well as the involvement of NHE1 activity during cellular polarization and migration (49). Lastly, ebselen was used as an inhibitor of NOX2 and peroxide scavenger (40, 41, 51), which also dampened cellular swelling and alkalization but not depolarization. It is noteworthy, that exposure to either BIX or ebselen did not largely alter most cellular functions in unstimulated cells, and, therefore, might be potential targets for clinical evaluations regarding the modulation of the PAF-induced thromboinflammatory response of neutrophils.

In addition to inducing an (electro-)physiological response in neutrophils, PAF elicited chemotactic activity, CD11b upregulation and CD62L downregulation, and PNC formation which is in accordance with previous studies (24, 25, 27, 52). This “platelet satelitism” of neutrophils is also found in several pathologies, including sepsis, augmenting neutrophil activity, including extravasation, neutrophil extracellular trap formation, and bacterial killing (53–56). It is tempting to speculate that inhibition of PNC formation, possibly caused by PAF release during sepsis (57, 58), might be a suitable target to modulate the thromboinflammatory response. For example, the striking relevance of PNC formation has been demonstrated in a murine model of hydrochloric acid-induced acute lung injury, in which blocking PNC formation significantly ameliorated organ dysfunction (59).

### PAF-induced (electro-)physiological Response in the Context of Severe Inflammation

During inflammation, neutrophils are required to migrate through various tissues and are exposed to varying levels of extracellular of extracellular proton concentrations. The pH of inflammatory body fluids, for example, abscesses, where neutrophils amass, is acidic (60–64). The experiment involving varying pH_e_ indicated that even under acidic pH_e_, neutrophils remain able to respond with a relative intracellular alkalization and cellular swelling despite a shift in their pH_i_ in relationship to the pH_e_. In addition, PAF-induced depolarization was increased in acidosis, potentially indicating elevated ROS generation, which has been demonstrated in a previous study (65).

Next, the effects of PAF on neutrophils were translationally investigated in two inflammatory environments. The previously described human whole blood model (36) of LPS-driven inflammation allows the investigation of blood physiology and immunity in an animal-free environment in accordance with the 3R principles (66). LPS exposure of whole blood altered the neutrophil phenotype as shown by CD11b and CD62L levels, confirming a sepsis-like phenotype that has been described in detail previously (67–71). LPS exposure did not alter the MP in comparison with unstimulated neutrophils and only augmented the PAF-induced response by a small amount. By contrast, a similar *in vitro* experiment comparing the depolarization of neutrophils with or without LPS exposure did report an increase in MP change, however, for fMLF (72). As observed in previous studies with neutrophils from septic humans and/or mice, exposure to LPS prompted an intracellular alkalization and swelling (14, 15, 73). This baseline drift in unstimulated cells was accompanied by a decrease in the PAF-induced response of granulocytes, possibly indicating ceiling effects. In general, the intracellular pH of neutrophils can rise beyond the LPS-induced shift in resting cells and/or the measurement method is capable of detecting larger shifts, arguing against a ceiling effect. Nevertheless, it is possible that neutrophils can only generate a certain alkaline shift, being limited by the pH_e_ that was not relevantly altered after LPS exposure in the whole blood model as reported earlier (36). LPS exposure in the whole blood model did not significantly change the sodium, potassium, or proton concentration, thus failing to explain the changes in alkalization or depolarization as reported previously (36).

Lastly, we analyzed the PAF-induced (electro-)physiological response in neutrophils before and during a well-characterized porcine model of polymicrobial sepsis (43–45). The clinical data confirmed a cardiocirculatory shock, indicating sepsis, also in corroboration with a significant hemoconcentration, suggesting a blood-organ barrier breakdown and subsequent hypovolemia. In the course of sepsis, the PAF-induced depolarization was significantly impaired, which confirmed findings in C5a-stimulated neutrophils after 3 h of porcine hemorrhagic shock (16). It is noteworthy, that there were no large shifts in extracellular ion concentrations and/or blood pH that could potentially explain this finding. Moreover, the PAF-mediated alkalization remained largely stable during sepsis, whereas cellular swelling was slightly increased. Also, the baseline of neutrophil pH_i_ and FSC-A did not shift (data not shown), which is in contrast to previous results in murine and/or human sepsis (14, 15, 73). These apparent differences in the findings within the two models might be explained by either species differences (human vs. pig), differences in the time points analyzed (1 h vs. 5.8 h ± 3.5), and/or regarding the pH_i_ with different fluorescent probes.

## Strengths and Limitations

Changes in the MP and pH_i_ of neutrophils were measured by fluorescent dyes *in vitro* yielding indirect results. However, direct measurement of MP by conventional patch-clamp in neutrophils is technically challenging and potentially results in artificial activation of the cells as well as a rapid exchange of the intracellular ion concentration (74, 75). Additionally, the results reported in the present work, including C5a as a positive control, are in agreement with previous studies (14–16). Besides not altering the neutrophil intracellular homeostasis, flow cytometry allows a high throughput of analyzed cells in comparison to the patch-clamp technique when simultaneously analyzing MP, intracellular pH, and cellular size as indicated by FSC-A. However, patch-clamp allows a higher temporal resolution in comparison to conventional flow cytometry. In the present work, we used continuous acquisition to partially compensate for the loss of temporal resolution. In addition, the current setup allowed the investigation of the PAF-induced cellular response in a physiologically environment at 37°C in the presence of ${\text{HCO}}_{3}^{-}$, the latter being involved in neutrophil ion channel activity (18, 76) and thereby should be included in measuring depolarization and intracellular alkalization. It is noteworthy that the ${\text{HCO}}_{3}^{-}$ containing buffer became slightly alkalotic, thereby likely explaining the alkaline shift in unstimulated neutrophils during the measurement period.

Next, we established a clear concentration-response relationship of the cellular response to PAF. However, we mainly used a concentration of 1 μM PAF (approximately 500 ng/ml). This concentration is higher than found in blood of septic neonates (2.3 ng/ml) (57). Anyhow, one must consider that PAF acts *in vivo* largely as a paracrine signaling molecule and neutrophils encounter PAF via other cells in the immediate vicinity (77). Many other cell types, including platelets and endothelial cells, which are in close proximity to neutrophils (e.g., by forming PNCs), secrete PAF when stimulated, for example, with thrombin, to promote neutrophil adhesion and transmigration (1, 2, 52, 78). In accordance, a study by Mitchell et al. that investigated fluid shear stress on neutrophils and effects of PAF applied a concentration of 1 μM to simulate a concentration neutrophils experience when coming into contact with endothelium (79). Further studies need to develop improved methods to quantify PAF and measure exact PAF levels locally and systemically during sepsis.

Lastly, we transferred the findings generated *in vitro* to the *ex vivo* whole blood model and the porcine sepsis model, which have several advantages and limitations. The whole blood model allowed the investigation of the PAF-induced response with or without LPS-induced inflammation in human blood in a well-defined system (36). In addition, changes of the neutrophil response to PAF were confirmed in an experimental model of polymicrobial sepsis (43–45) with a clinically relevant pathophysiological reaction. Interestingly, the results of both models varied. This might arise because of several reasons: On the one hand, there might be notable interspecies differences. On the other hand, the assay itself differed between the two models regarding the pH_i_, because porcine neutrophils had to be analyzed with a different dye. Furthermore, there are different kinetics of the inflammatory response to consider: In the whole blood model of endotoxemia, there was an initial stimulation with LPS resulting in a rapid inflammatory response, while the addition of bacteria in the porcine model triggered a gradually increasing inflammation. In addition, the duration of the period analyzed and/or the inflammatory response elicited by LPS vs. fecal inoculation might explain the different results. In the whole blood model, it is difficult to analyze neutrophils in a long-term investigation, because glucose levels decline while in parallel lactate levels and acidosis increase, resulting in an unphysiological environment beyond 1 h. These issues were circumvented and thus indicated the application of the porcine sepsis model. Moreover, the lack of the presence of the interaction of neutrophils with other organs may play an important role. Another important aspect to consider regarding the porcine sepsis model is that neutrophils were isolated at the onset of sepsis as indicated by a drop of mean arterial blood pressure but prior to severe organ dysfunction. The rationale for this was to increase the sample size, since the current experiments were embedded in an interventional trial, that started after the beginning of sepsis (reduction of animal numbers). Also, the onset of sepsis marked the initiation of a resuscitation therapy, which may have further altered the response of neutrophils and likely ameliorated the degree of the inflammatory response such as lactate acidosis. Taken together, this allowed the investigation of neutrophil dysfunction as an early hallmark of the beginning of sepsis. Further research needs to explore these findings in a non-resuscitated model of sepsis and/or during a more pronounced inflammatory response including systemic acidosis, which may reveal further changes also in pH_i_ and FSC. Moreover, the findings need to be confirmed in patients with sepsis. Various causes of a potential neutrophil desensitization to stimulation with PAF during sepsis should be investigated, for example, a receptor downregulation of PAF, a previous stimulation by PAF *in vivo*, and/or a defect in PAF-signaling in sepsis. Notably, both models offer a distinct advantage investigating neutrophil pathophysiology during sepsis: Analyzing patients with sepsis does hardly allow firm assumptions regarding the onset of the infection, which was clearly defined in the model systems analyzed in this work.

## Conclusion

Platelet-activating factor (PAF) is an important activator of neutrophils triggering depolarization, intracellular alkalization, and changes in cellular size. The PAF-induced response was demonstrated to be altered in an *ex vivo* model of endotoxemia and at the onset of porcine experimental sepsis, which might be an early hallmark of innate immune dysfunction during sepsis. Further studies are warranted to elucidate the association between the modulated (electro-)physiological response and cellular effector function. Additionally, translational efforts are needed to explore the therapeutic and diagnostic potential of these findings, for example, by measuring the neutrophil response at the patient's bedside and/or by targeting the involved ion channels, including NHE1, to reduce overwhelming PAF-induced neutrophil activation.

## Data Availability Statement

The original contributions presented in the study are included in the article/Supplementary Material, further inquiries can be directed to the corresponding author.

## Ethics Statement

The studies involving human volunteers were reviewed and approved by the Local Independent Ethics Committee (number 459/18; 94/14) of the University of Ulm. The participants provided their written informed consent to participate in this study. The animal study was reviewed and approved by the Federal Authorities for Animal Research (#1362, Tuebingen, Germany) as well as the Animal Care Committee of the University of Ulm.

## Author Contributions

DM and MH-L: conceptualization and supervision. SH, SB, and DM: data curation and formal analysis. SH, SB, PR, AH, and DM: methodology. SH, AS, and DM: validation and visualization. SH and DM: writing—original draft. All authors contributed to the article and approved the submitted version.

## Conflict of Interest

The authors declare that the research was conducted in the absence of any commercial or financial relationships that could be construed as a potential conflict of interest.

## Supplementary Material

Supplement 1 PAF-mediated changes in the response of neutrophils were concentration-dependent. Concentration-response curves were calculated for the following parameters after stimulation with PAF (1 nM−1 μM) at the time point of maximal stimulation as indicated: **(A)** FSC-A (10 min), **(B)** SSC-A (10 min), **(C)** membrane potential (1 min), **(D)** intracellular pH (5 min), **(E)** ROS generation (10 min), **(F)** formation of platelet–neutrophil complexes measured by flow cytometry (15 min), **(G)** L-selectin (CD62L) surface expression (15 min), and **(H)** integrin alpha M (CD11b) surface expression (15 min). Data are mean ± SD (*n* = 5–10).

Supplement 2 Tabular overview of the relative PAF-induced effects on human neutrophils and gender analysis at the point of a (near-)maximal response during the first hour. FSC-A, forward scatter area; SSC-A, side scatter area; MP, membrane potential; pH_i_, intracellular pH; ROS, generation of reactive oxygen species; CTX, chemotaxis; CD62L, L-selectin surface expression downregulation; CD11b, integrin alpha M surface expression upregulation. Data are mean ± standard deviation (*n* = 5 – 10). ^*^*p* < 0.05, ^**^*p* < 0.01, ^***^*p* < 0.001, for comparing PAF vs. CTRL: MP Wilcoxon signed-rank test, for all other parameters Wilcoxon matched-pairs signed rank test. For the comparison of neutrophils from male or female donors: Mann-Whitney test.

Supplement 3 Modulation of neutrophil parameters by pharmacological inhibitors without further stimulation. The extent of these effects was compared to the response induced by 1 μM PAF. Control = 0%, PAF-mediated effect = 100%. Boxes outside the defined range were colored gray. For calculation see Supplement 4. FSC-A, forward scatter area; SSC-A, side scatter area; MP, membrane potential; pH_i_, intracellular pH; ROS, generation of reactive oxygen species; CTX, chemotaxis; CD62L, L-selectin surface expression downregulation; CD11b, Integrin alpha M surface expression upregulation. Data are mean (*n* = 5–10).

Supplement 4 Calculations for **(A)** Figure 4 and **(B)** the Heatmap in Supplement 3; F, mean fluorescence intensity.
