# Supplementary figures and images for: Activation of Neutrophil Granulocytes by Platelet-Activating Factor Is Impaired During Experimental Sepsis
Source: Front Immunol. 2021 Mar 16;12:642867. doi: 10.3389/fimmu.2021.642867 (PMC8007865; doi:10.3389/fimmu.2021.642867)

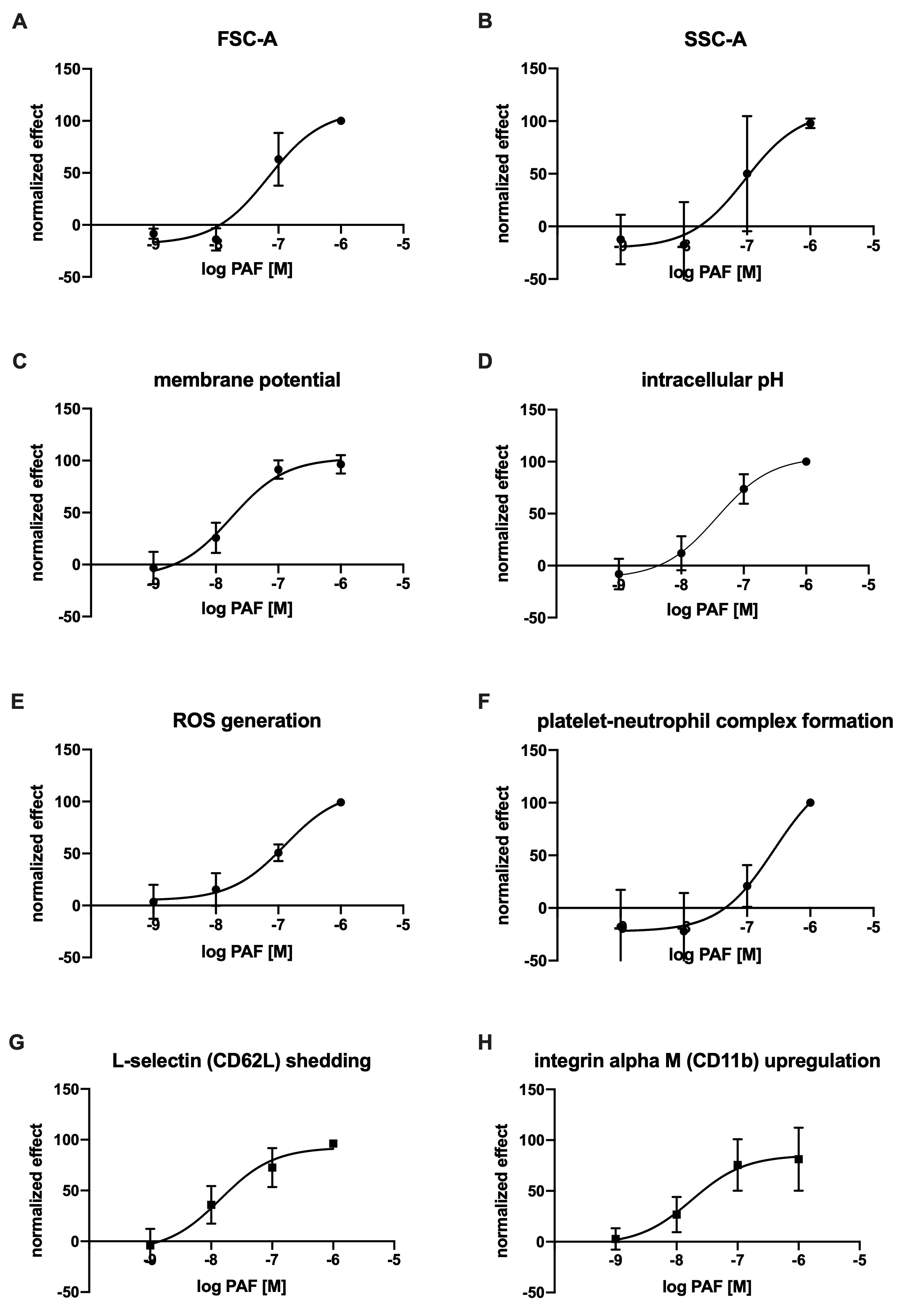

Supplement: Supplement 1 — PAF-mediated changes in the response of neutrophils were concentration-dependent. Concentration-response curves were calculated for the following parameters after stimulation with PAF (1 nM−1 μM) at the time point of maximal stimulation as indicated: (A) FSC-A (10 min), (B) SSC-A (10 min), (C) membrane potential (1 min), (D) intracellular pH (5 min), (E) ROS generation (10 min), (F) formation of platelet–neutrophil complexes measured by flow cytometry (15 min), (G) L-selectin (CD62L) surface expression (15 min), and (H) integrin alpha M (CD11b) surface expression (15 min). Data are mean ± SD (n = 5–10). [file Image_1.jpg]

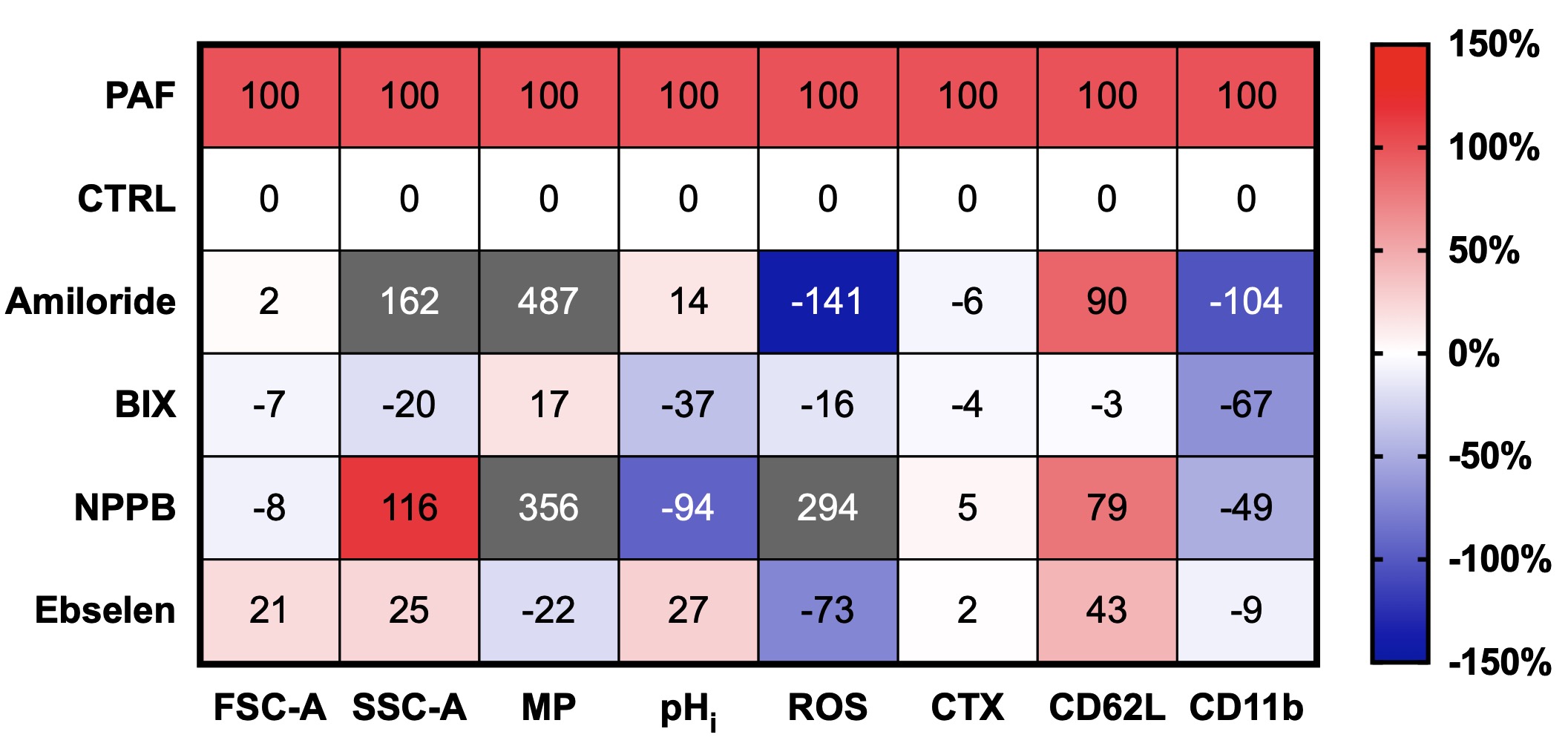

Supplement: Supplement 3 — Modulation of neutrophil parameters by pharmacological inhibitors without further stimulation. The extent of these effects was compared to the response induced by 1 μM PAF. Control = 0%, PAF-mediated effect = 100%. Boxes outside the defined range were colored gray. For calculation see Supplement 4. FSC-A, forward scatter area; SSC-A, side scatter area; MP, membrane potential; pHi, intracellular pH; ROS, generation of reactive oxygen species; CTX, chemotaxis; CD62L, L-selectin surface expression downregulation; CD11b, Integrin alpha M surface expression upregulation. Data are mean (n = 5–10). [file Image_3.jpg]
