# Supplementary material for: Activation of Neutrophil Granulocytes by Platelet-Activating Factor Is Impaired During Experimental Sepsis
Source: Front Immunol. 2021 Mar 16;12:642867. doi: 10.3389/fimmu.2021.642867 (PMC8007865; doi:10.3389/fimmu.2021.642867)
Supplement: Supplement 2 — Tabular overview of the relative PAF-induced effects on human neutrophils and gender analysis at the point of a (near-)maximal response during the first hour. FSC-A, forward scatter area; SSC-A, side scatter area; MP, membrane potential; pHi, intracellular pH; ROS, generation of reactive oxygen species; CTX, chemotaxis; CD62L, L-selectin surface expression downregulation; CD11b, integrin alpha M surface expression upregulation. Data are mean ± standard deviation (n = 5 – 10). *p < 0.05, **p < 0.01, ***p < 0.001, for comparing PAF vs. CTRL: MP Wilcoxon signed-rank test, for all other parameters Wilcoxon matched-pairs signed rank test. For the comparison of neutrophils from male or female donors: Mann-Whitney test. [file Image_2.pdf]

Supplement 2: Tabular overview of the relative PAF-induced effects on human neutrophils and gender analysis at the point of a (near-)maximal response during the first hour. FSC-A = forward scatter area, SSC-A = side scatter area, MP = membrane potential, pH_i = intracellular pH, ROS = generation of reactive oxygen species, CTX = chemotaxis, CD62L = L-selectin surface expression downregulation, CD11b = integrin alpha M surface expression upregulation. Data are mean ± standard deviation (n = 5 – 10)

Parameter	PAF vs. CTRL	PAF 1 µM	Unit	Reported measurement point	PAF ♂	PAF ♀	♂ vs. ♀
**FSC-A**	**	63 ± 16	% PAF vs. ctrl	10 min	66 ± 21	60 ± 6	$p = 0.9$
**SSC-A**	***	13 ± 4	% PAF vs. ctrl	10 min	13 ± 3	13 ± 4	$p = 0.6$
**MP**	***	+12.2 ± 4.1	mV	1 min	10.8 ± 2.4	13.9 ± 5.0	$p = 0.2$
**pH_i**	***	0.45 ± 0.07	delta	5 min	0.45 ± 0.08	0.45 ± 0.07	$p = 0.5$
**ROS**	***	26 ± 11	% PAF vs. ctrl	10 min	26 ± 6	28 ± 17	$p = 0.9$
**CTX**	*	15.8 ± 9.7	fold increase	30 min	14.2 ± 7.6	18.8 ± 12.3	$p = 0.8$
**CD62L**	**	−87 % ± 10 %	% PAF vs. ctrl	10 min	−90-6 ± 2.8	−83.3 ± 13.5	$p > 0.9$
**CD11b**	**	63.3 ± 41.9	% PAF vs. ctrl	10 min	50.0 ± 24.5	80.0 ± 52.0	$p = 0.4$
