# Supplementary material for: Activation of Neutrophil Granulocytes by Platelet-Activating Factor Is Impaired During Experimental Sepsis
Source: Front Immunol. 2021 Mar 16;12:642867. doi: 10.3389/fimmu.2021.642867 (PMC8007865; doi:10.3389/fimmu.2021.642867)
Supplement: Supplement 4 — Calculations for (A) Figure 4 and (B) the Heatmap in Supplement 3; F, mean fluorescence intensity. [file Image_4.pdf]

A

$$x = \frac{F_{(\text{Inhibitor+PAF})} - F_{(\text{Inhibitor+CTRL})}}{F_{(\text{CTRL+PAF})} - F_{(\text{CTRL+CTRL})}} - 1$$

B

$$x = \frac{F_{(\text{Inhibitor+CTRL})} - F_{(\text{CTRL+CTRL})}}{F_{(\text{CTRL+PAF})} - F_{(\text{CTRL+CTRL})}}$$

Supplement 4: Calculations for Figure 4 (**A**) and the heatmap in Supplement 3 (**B**). F = mean fluorescence intensity
